# Comparison of Different Laboratory Tests to Identify “Aspirin Resistance” and Risk of Vascular Events among Ischaemic Stroke Patients: A Double-Blind Study

**DOI:** 10.3390/jcdd9050156

**Published:** 2022-05-12

**Authors:** Narayanaswamy Venketasubramanian, Sherwin Joy Agustin, Jorge L. Padilla, Maricar P. Yumul, Christina Sum, Sze Haur Lee, Kuperan Ponnudurai, Robert N. Gan

**Affiliations:** 1 Raffles Neuroscience Centre, Raffles Hospital, Singapore 188770, Singapore; 2 Research Department, National Neuroscience Institute, Singapore 188770, Singapore; sherwinjoymd@yahoo.com; 3 Department of Medicine, Cotabato Regional and Medical Center, Cotabato 9600, Philippines; hor_he@yahoo.com; 4 Department of Neurology and Psychiatry, University of Santo Tomas Hospital, Manila 1015, Philippines; maricar.p.yumul@gmail.com; 5 Department of Laboratory Medicine, Tan Tock Seng Hospital, Singapore 188770, Singapore; christina_sum@ttsh.com.sg (C.S.); ponnudurai_kuperan@ttsh.com.sg (K.P.); 6 Department of Neurology, National Neuroscience Institute, Tan Tock Seng Campus, Singapore 188770, Singapore; sze_haur_lee@nni.com.sg; 7 Medical Affairs, Moleac Singapore, Pte Ltd., Singapore 188770, Singapore; robert_gan2@hotmail.com

**Keywords:** aspirin resistance, stroke, outcome

## Abstract

“Aspirin resistance” (AR) is associated with increased risk of vascular events. We aimed to compare different platelet function tests used in identifying AR and assess their implications on clinical outcome. We performed platelet aggregation studies on non-cardioembolic ischaemic stroke patients taking aspirin 100 mg/day and 30 non-stroke controls. Data were collected on demographics, vascular risk factors, and concomitant medications. Cut-offs for AR were (1) light transmission aggregometry (LTA) of ≥20% using arachidonic acid (AA), ≥70% using ADP, or ≥60% using collagen; and (2) VerifyNow^®^ assay ≥ 550 ARU. Telephone follow-ups were conducted by study staff blinded to AR status to ascertain the occurrence of vascular outcomes (stroke, myocardial infarction, amputation, death). A total of 113 patients were recruited, mean age 65 ± 8 years, 47% women, 45 ± 15 days from index stroke. 50 (44.3%, 95% CI 34.9–53.9) had AR on at least 1 test. Frequency of AR varied from 0% to 39% depending on method used and first vs. recurrent stroke. There were strong correlations between LTA AA, VerifyNow^®^ and Multiplate^®^ ASPItest (r = 0.7457–0.8893), but fair to poor correlation between LTA collagen and Multiplate^®^ COLtest (r = 0.5887) and between LTA ADP and Multiplate^®^ ADPtest (r = 0.0899). Of 103 patients with a mean follow up of 801 ± 249 days, 10 (9.7%) had vascular outcomes, of which six had AR by LTA-ADP. AR by LTA-ADP is associated with increased risk of vascular outcome (*p* = 0.034). Identification of AR is not consistent across different platelet function tests. LTA of ≥70% using 10 µM ADP in post-stroke patients taking aspirin is associated with increased risk of vascular outcome.

## 1. Introduction

Use of anti-platelet drugs reduces the risk of recurrent stroke, myocardial infarction or vascular death after an acute ischaemic stroke (IS) or transient ischaemic attack (TIA) [1]. Aspirin, an irreversible inhibitor of platelet prostaglandin synthase activity and the most widely used antiplatelet for secondary prevention, at the dose of <150 mg/day, reduces the risk of all stroke by 21% (0.79 (95 % CI, 0.66 to 0.95) [2]. Unfortunately, 10−20% of patients develop a recurrent vascular event despite taking antiplatelet medications [3]. The term ‘aspirin resistance (AR)’ has been introduced to describe these situations when clinical or ex vivo effects of aspirin are less than expected [4]. Total prevention of stroke recurrence may not be possible due to the natural history of the disease and the effect of non-modifiable risk factors such as aging. Other possible reasons for ‘aspirin failure’ include inappropriate prescription (e.g., for cardioembolism), reduced adherence to treatment, inadequate dose of aspirin, use of poorly absorbed forms of aspirin, intake of drugs that may interact with aspirin and thus inhibit its mechanism of action (e.g., certain nonsteroidal anti-inflammatory drugs), confounding conditions such as diabetes mellitus, genetic polymorphisms, alternate pathways of platelet activation, aspirin-insensitive thromboxane biosynthesis, increased platelet turnover, etc. [3,5,6,7].

Attention has focused on laboratory-based “aspirin resistance (AR)” as one reason for such aspirin failure that may require new antiplatelet strategies. A systematic review and network meta-analysis reported that among patients with IS/TIA, antiplatelet high on-treatment platelet reactivity (a-HTPR) was 3 to 65% with aspirin [8]. Overall, there was a higher risk of the composite primary outcome of stroke/TIA, myocardial infarction, vascular death (OR 2.93, 95% CI 1.90–4.51) and recurrent ischaemic stroke/TIA (OR 2.43, 95% CI 1.51–3.91) in patients with vs. those without a-HTPR on any antiplatelet regimen. These risks were also more than two-fold higher in patients with vs. those without ‘aspirin-HTPR’. The risk of severe stroke was higher in those with vs. without a-HTPR (OR 2.65, 95% CI 1.00–7.01). This makes identification of this subgroup of patients with aspirin resistance important. It is, however, unclear which assay best detects this. The available tests include testing whole blood usually at point of care (including whole blood aggregometry, platelet counting, platelet function tests, flow cytometry), some require sample preparation (e.g., light transmission aggregometry, serum or plasma thromboxane B2 measurement), and some test the urine (e.g., urine thromboxane B2 or its metabolite 11-dehydro thromboxane B2) [9]. The role of platelet function tests to guide stroke therapy remains unclear [10]. However, some feel it is reasonable to test for aspirin resistance [11].

We, therefore, primarily aimed to estimate the frequency of AR in our population of ischaemic stroke patients using different laboratory methods of assessing platelet function. A secondary objective was to correlate the findings of AR by different methods with the occurrence of composite vascular outcome defined as death, recurrent stroke, heart attack, and/or amputation.

## 2. Materials and Methods

### 2.1. Subject Selection

We recruited patients ≥50 years old with first or recurrent non-cardioembolic ischaemic stroke and who were prescribed aspirin 100 mg/day for secondary prevention. Recurrent stroke must have had occurred while taking aspirin prescribed for a previous stroke. Exclusions were cardioembolic strokes, cases with clear indication for anticoagulation, intake of NSAID or antiplatelets other than aspirin in the preceding two weeks, known medical contraindication to aspirin, pregnancy, fever in the preceding 2 weeks, or inability to comply with the protocol.

After obtaining informed consent, data on demographics, historical vascular risk factors (i.e., hypertension, diabetes, hyperlipidemia, coronary artery disease, smoking, alcohol intake), recent intake of medications and supplements, and compliance to aspirin were collected through interview and review of medical records. Hypertension was defined as having been previously diagnosed by a physician to have hypertension, or taking blood pressure lowering medication, or a blood pressure ≥ 140/90 mmHg. Diabetes mellitus was defined as having been previously diagnosed by a physician to have diabetes mellitus, or taking blood glucose lowering medication, or fasting blood glucose ≥ 7 mmol/L. Hyperlipidaemia was defined as having been previously diagnosed by a physician to have hyperlipidaemia, or taking lipid lowering medication, or a fasting LDL-cholesterol ≥ 4.1 mmol/L. Coronary artery disease was defined as having been previously diagnosed by a physician to have coronary artery disease, angina, myocardial infarction, or having had procedures for coronary artery stenosis. Smoking was diagnosed as having smoked in the 30 days prior to the study participation. Alcohol intake was diagnosed as having had alcohol in the 30 days prior to study participation. Index stroke status (first or recurrent) and stroke mechanism based on the TOAST classification were recorded [12].

Thirty control subjects ≥50 years old with no history of stroke, not on aspirin, and had none of the exclusion criteria were recruited by advertisement for the laboratory correlation study and had the same set of procedures performed. They were not matched with patients for age, sex or vascular risk factors so as to allow inclusion of these potential confounders in the analysis. The inclusion of the control arm was to determine the typical results using the different platelet assessment methods among subjects not taking aspirin.

### 2.2. Laboratory Tests

We collected morning fasting blood samples 30 to 60 days after index stroke. Blood was taken before the dose of aspirin that day to allow AR testing at trough level. Samples were analyzed within 30 min for:

#### 2.2.1. Light Transmission Aggregometry (LTA)

Platelet aggregation responses were recorded from optical aggregometer (Chrono-log, Havertown, PA, USA) as maximal percent change in light transmission compared to baseline after addition of agonist: 0.5 mM arachidonic acid (AA), 2 µg/ml collagen, or 10 µM ADP (Chrono-log, Havertown, PA, USA).

#### 2.2.2. Whole Blood Turbidimetric-Based Aggregometry (VerifyNow^®^ System, Accumetrics, San Diego, CA, USA)

Whole blood samples were introduced into the aspirin assay device that uses arachidonic acid to activate platelets. The instrument assesses platelet function based on binding of activated platelets to lyophilized preparation of human fibrinogen by measuring the increase in optical signal caused by platelet aggregation and converts results into “aspirin resistance units (ARU).”

#### 2.2.3. Whole Blood Multiple Electrode Impedance Aggregometry (Multiplate^®^ Platelet Function Analyzer, Dynabyte Medical, Munich, through Dyamed Biotech, Singapore)

Diluted (50%) whole blood samples were pipetted into disposable test cells that contain two impedance sensors, each consisting of two parallel highly conductive copper electrodes. Agonist solutions AA (ASPItest), collagen (COLtest), or ADP (ADPtest) were added and platelet aggregation and attachment onto the metal sensors were continuously detected by measuring change in electrical resistance between the two electrode wires. The increase in impedance was transformed to arbitrary “aggregation units (AU)” and plotted against time. Parameters calculated are the area under the aggregation curve (AUC), aggregation, and velocity.

### 2.3. Definition of Aspirin Resistance (AR)

Following previously published reports, we defined AR as LTA of ≥20% on AA, ≥70% on ADP, ≥60% on collagen, or ≥550 ARU on VerifyNow^®^. As there is no consensus on what cut-offs (reported in AUC) define “AR” by Multiplate, mean values were used to allow comparison with the other methods.

### 2.4. Subject Follow-Up

A study staff blinded to the laboratory results contacted participants by telephone to inquire about clinical outcomes (recurrent stroke, myocardial infarction, amputation or death) using a standard questionnaire. Data on use of aspirin and other medications, survival (dead or alive), hospitalization since last contact, major bleeding (intracranial, haemodynamic compromise or requiring blood transfusion) [13]. Participants were not informed of their AR status. Medical records, whenever applicable, were reviewed for confirmation.

### 2.5. Statistical Analysis

Calculation using 20% estimated prevalence and 95% confidence interval of 10–30% gave a sample size of 62 (© Simple Interactive Statistical Analysis) for each stroke group. Comparisons of means were performed using *t*-test and ANOVA and comparisons of proportions were analyzed by χ^2^-test, using Fisher exact when necessary. Correlation analysis was used to compare between platelet function tests. Kaplan–Meier was used to estimate and compare the risks of vascular outcomes in different groups. Cox regression was used to adjust for potential confounders.

This study was approved by the institutional review board and was supported by the National Medical Research Council of Singapore.

## 3. Results

### 3.1. Participant Characteristics

One hundred and thirteen post-stroke patients (mean age 65 ± 8 years; women 47%) and 30 controls were recruited (Table 1). All patients had at least one stroke risk factor: 16 (14%) had one, 41 (36%) had two, 47 (42%) had three, 9 (8%) had four, and none had >4 risk factors. No patient missed taking aspirin more than once a week in the two weeks preceding the blood tests.

### 3.2. Aspirin Resistance

**Figure 1:**
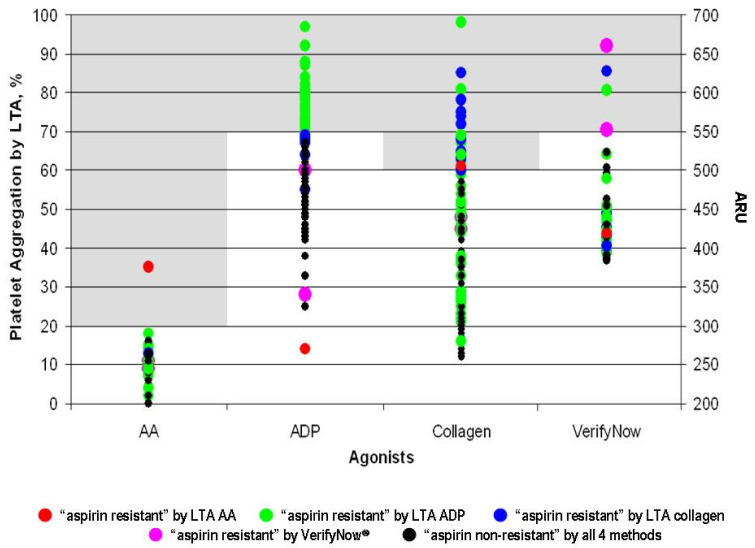
Comparison of different platelet aggregation tests in identifying “aspirin resistance”. Shaded area corresponds to cut-off for “aspirin resistance” for each test used.

Among the stroke patients, 50/113 (44.3%, 95% CI 34.9–53.9) were AR positive on at least one test. The overall frequency of AR among post-stroke patients ranged from 0 to 39%, depending on method used and first vs. recurrent stroke (Table 2). Identification of AR in a particular patient is inconsistent across different laboratory methods, i.e., a patient with AR by one method may not be so using another method (Figure 1). Comparing the different laboratory methods, we found strong correlations between LTA AA, VerifyNow^®^ and Multiplate^®^ ASPItest (r = 0.7457–0.8893), but fair to poor correlation between LTA collagen and Multiplate^®^ COLtest (r = 0.5887) and between LTA ADP and Multiplate^®^ ADPtest (r = 0.0899).

We found no clear relationship between laboratory AR or degree of platelet aggregation and age, gender, ethnicity, stroke status (first versus recurrent), history of hypertension, diabetes mellitus, hyperlipidemia, coronary artery disease, smoking, number of risk factors.

### 3.3. Outcome

We were able to follow up 132 of 143 subjects (92%; 103/113 post-stroke patients; 29/30 controls) with mean follow-up of 801 ± 249 days for post-stroke patients and 1087 ± 134 days for controls. Among post-stroke patients, one had major bleeding. Ten (9.7%; first stroke 5, recurrent stroke 5) had clinical vascular outcome occurring 462 ± 347 (mean) days from index stroke, (7.9%/yr). Six of them were AR by LTA ADP (Table 3). None of the controls had any outcome.

**Figure 2:**
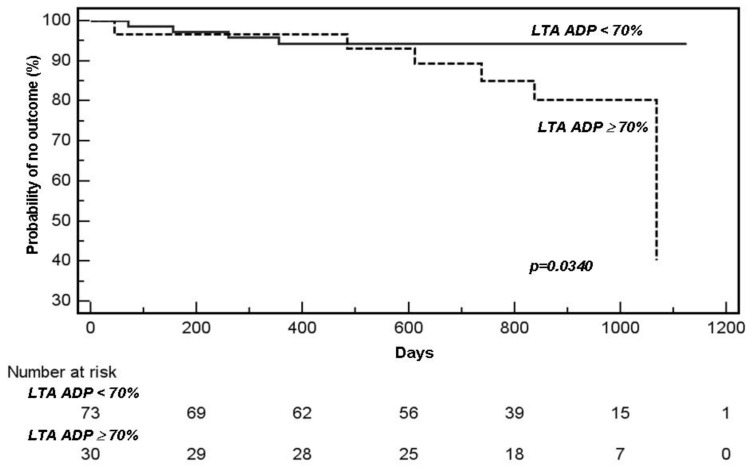
Kaplan–Meier curves of group considered “aspirin resistant” versus not “aspirin resistant” by light transmission aggregometry (LTA) using ADP.

We found an increased, but not statistically significant, risk of having a vascular outcome in the group identified as AR by any of the laboratory tests compared to those identified as not being AR in any of the tests. AR by LTA AA, LTA collagen, or VerifyNow^®^ was not associated with increased risk of vascular outcome, although the numbers for LTA AA and VerifyNow^®^ were very small. However, we found a significantly increased risk in the group identified as AR by LTA ADP compared to the non-AR group (Figure 2), even after adjusting for age, gender, ethnicity, number of risk factors, regular follow-up with a physician and compliance to aspirin (*p* = 0.034).

## 4. Discussion

Our study showed that AR is found in 44.3% of our IS patients, with a range of 0 to 39%, depending on the type of test used and first vs. recurrent stroke. There were strong correlations between some of the tests, but not among others. Almost 10% had a vascular outcome on follow-up, with a significant risk seen among patients with AR by LTA-ADP.

The finding of AR of 44.3% in our study is consistent with the 3 to 65% reported in the systematic review and network meta-analysis [8]. Studies in other Asian ischaemic stroke populations detected AR in 17.9 to 20.4% among Chinese studies [14,15,16,17,18], and 3.1% in an Indian study [19]. It was 14% in a Malaysian study comprising Chinese, Indians and Malays [20].

Increased doses of aspirin have a dose-dependent effect on platelet aggregability, based on studies in stroke patients using daily doses of 40 mg to 1.3 g [21,22]. Our study used a fixed dose of 100 mg, which is within the range recommended by local and international guidelines [10,23].

We found good correlation between different methods that assess AA-induced platelet aggregation (LTA AA, VerifyNow^®^ and Multiplate^®^ ASPItest), but fair to poor correlation for methods assessing ADP- and collagen-induced aggregation. This lack of consistent finding of AR by differing tests in the same stroke patient have also been reported by others [24,25,26,27]. Good correlation was reported between LTA and thromboelastography (TEG) in one study [26], and VerifyNow^®^ and TEG in another [27]. The wide variability in the reported prevalence of AR may be reflective of the different laboratory methods and parameters used. Our study findings are similar to that by Lordkipanidzé et al. which showed poor correlation and agreement between six platelet function tests for AR among patients with stable coronary artery disease [28].

We found an increased, but not statistically significant risk, of having a vascular outcome in the group identified as AR by any of the laboratory tests, consistent with the systematic review and network meta-analysis showing an increased risk. Increased risk has also been found in studies in China [14,15,16,17,18]. However, the best predictive AR test is still unknown. Our study showed that AR detected by LTA-ADP was the best predictor of recurrent stroke, myocardial infarction and death among our cohort of ischaemic stroke patients. The proportion of stroke recurrence and composite events in patients with AR using ADP as assessed by PL-12 was higher than VerifyNow and TEG in one study [27]; cardiovascular events and death were found among those with AR diagnosed by urine dTXB2 measurement rather than VerifyNow [25]. Reproducibility of AR on re-test a year later has been shown to be poor [24].

For a laboratory measure of AR to be clinically useful, it should (1) have consistent and independent association with recurrent vascular events in spite of aspirin intake, (2) be standardized and valid, (3) alter clinical management, and (4) have favorable benefit-to-risk/cost profile [29]. In our study, we found that simple detection of non-inhibition of ADP-mediated platelet aggregation among patients on aspirin may explain, at least, some “aspirin failures” who develop vascular events despite usage of aspirin and adequate blocking of arachidonic acid pathway. This may be consistent with a much earlier finding of synergism between arachidonic acid and other agonists despite use of aspirin and reports of single nucleotide polymorphisms in the ADP gene in AR patients [30,31]. This has obvious implications on treatment and may support the hypothesis (that may be tested in a clinical trial) that such patients may benefit from other additional antiplatelets that either inhibit ADP-mediated platelet activity or through other anti-platelet pathways. Of our 36 patients who had AR by LTA-ADP, 21 (58.3%) were diabetic. Increased platelet turnover as evidenced by the presence of young hyper-reactive reticulated platelets in the circulation is seen in diabetics and acute myocardial infarction [32] that could contribute to the observed AR. This may support fractioning of aspirin administration instead of merely increasing the daily dose of aspirin, e.g., twice daily dosing with aspirin, particularly in diabetics—this approach has been shown to significantly decrease mean TxB2 levels compared to once-a-day dosing [33]. Due to the expense and tediousness of performing traditional LTA, finding a simple point-of-care assessment of ADP-mediated platelet aggregation that correlates well with LTA shall remarkably help in the design of such future studies.

Our study has several limitations. Our sample size was small—a larger sample size may have detected more significant findings. We were only able to recruit 51 of the target of 62 patients with recurrent stroke—this was because recurrent stroke patients were fewer in number than first stroke patients; still, we feel recruiting 51/62 (=82.2%) of target still provides meaningful data. Controls were not matched for age, sex or vascular risk factors; they only had to be >50 years old and stroke-free—while this led to differences in baseline vascular risk factors, this is not unexpected as older individuals with stroke on aspirin would more likely have vascular risk factors, as our study has shown. All our patients were only on 100 mg daily of aspirin—patients on higher doses of aspirin may exhibit less laboratory “aspirin resistance” and may have less risk of a recurrent vascular event. We did not use other platelet activity tests, or different concentrations of agonists in our study as we did not have available these other tests at the time of study—other tests may have given additional insights into the prevalence of AR and subsequent vascular risk. We also did not repeat the platelet function tests in our cohort to assess for long-term progressive loss of inhibition by aspirin—delayed development of AR may have explained the occurrence of vascular events [34,35]. Still, our study involves a well-characterized cohort of ischaemic stroke patients on the guideline-recommended and stable dose of aspirin, patients compliant with medication, a control group, a long follow-up with low drop-out rate, patients and assessors blinded to AR status, outcome cross-checked against medical records, and use of multiple tests for AR.

## 5. Conclusions

Our study detected AR at a comparable frequency with other studies; we found that identification of AR and its frequency depends on the laboratory parameter used and may not necessarily be consistent across the different methods of testing platelet activity, and that a residual LTA of ≥70% using 10 µM ADP among post-stroke patients on aspirin is associated with an increased risk of vascular outcome. Studies with larger sample size should be performed to confirm the findings of our pilot study.

## Figures and Tables

**Table 1:** Patient demographics according to stroke status.

	First Stroke (F) (*n* = 61)	Recurrent Stroke (R) (*n* = 52)	Control (C) (*n* = 30)		*p*-Value	
Age, mean ± SD (years) *	64 ± 8	67 ± 8	57 ± 4	F vs. R 0.18	F vs. C 0.0004	R vs. C <0.0001
Female, *n* (%)	31 (51%)	22 (42%)	15 (50%)	0.37	0.94	0.50
Ethnicity, *n* (%)						
Chinese	54 (89%)	43 (83%)	30 (100%)	0.38	0.05	0.02
Malay	3 (5%)	8 (15%)	0 (0%)	0.06	0.55	0.02
Indian	4 (7 %)	0 (0%)	0 (0%)	0.06	0.15	1
Other	0 (0%)	1 (2%)	0 (0%)	1	1	1
Risk factors, *n* (%)						
No risk factor	0 (0%)	0 (0%)	10 (33%)	1	<0.0001	<0.0001
Hypertension	45 (74%)	44 (85%)	11 (37%)	0.16	0.0006	<0.0001
Diabetes mellitus	31 (51%)	32 (62%)	8 (27%)	0.25	0.03	0.002
Hyperlipidemia	56 (92%)	47 (90%)	11 (37%)	0.79	<0.0001	<0.0001
Coronary artery disease	2 (3%)	4 (8%)	0 (0%)	0.30	0.55	0.12
Smoking	6 (10%)	8 (15%)	2 (7%)	0.37	0.62	0.25
Alcohol	0 (0%)	0 (0%)	2 (7%)	1	0.11	0.13
Stroke subtype, *n* (%) **						
Atherosclerotic (extracranial)	6 (10%)	5 (10%)	n/a	0.97		
Atherosclerotic (intracranial)	14 (23%)	33 (63%)	n/a	<0.001		
Lacunar/small vessel disease	32 (52%)	13 (25%)	n/a	0.003		
Cryptogenic	9 (15%)	1 (2%)	n/a	0.02		
Concomitant medications, *n* (%)						
Blood pressure lowering	26 (43%)	24 (46%)	11 (37%)	0.71	0.59	0.41
Lipid lowering	56 (92%)	49 (94%)	10 (33%)	0.62	<0.0001	<0.0001
Hypoglycemic	26 (43%)	25 (48%)	6 (20%)	0.56	0.03	0.01
Supplements in past 2 weeks, *n* (%)						
Glucosamine tablets	1 (2%)	1 (2%)	3 (10%)	1	0.07	0.10
Calcium tablets	0 (0%)	0 (0%)	5 (17%)	1	0.001	0.002
Red wine	0 (0%)	1 (2%)	8 (27%)	1	<0.0001	0.0006
Grape juice	0 (0%)	1 (2%)	2 (7%)	1	0.11	0.27
Vitamin E	1 (2%)	0 (0%)	4 (13%)	1	0.02	0.007
Gingko	1 (2%)	1 (2%)	3 (10%)	1	0.07	0.10
Ginseng	4 (7%)	1 (2%)	2 (7%)	0.23	0.98	0.27
Garlic tablets	3 (5%)	0 (0%)	2 (7%)	0.11	0.73	0.13
Traditional Chinese medicine	6 (10%)	6 (12%)	2 (7%)	0.77	0.62	0.13

* *p* < 0.05; ** *p* < 0.001.

**Table 2:** Results of platelet function testing in the different groups.

Laboratory Test	Post-Stroke Patients			Control (*n* = 30)
	All (*n* = 113)	First (*n* = 61)	Recurrent (*n* = 52)	
Light transmission aggregometry	*n* = 113	*n* = 61	*n* = 52	*n* = 30
Arachidonic acid 0.5 mM				
Mean ± SD, %	12 ± 4	10 ± 3	13 ± 4	91 ± 6
≥20% aggregation *, n (%)	*1 (1%)*	*0 (0%)*	*1 (2%)*	*30 (100%)*
ADP 10 µM				
Mean ± SD, %	62 ± 15	62 ± 18	63 ± 11	83 ± 12
≥70% aggregation *, n (%)	*36 (32%)*	*24 (39%)*	*12 (23%)*	*26 (87%)*
Collagen 2 ug/ml				
Mean ± SD, %	41 ± 19	38 ± 19	44 ± 18	88 ± 8
≥60% aggregation *, n (%)	*22 (19%)*	*9 (15%)*	*13 (25%)*	*30 (100%)*
VerifyNow^®^	*n* = 112	*n* = 61	*n* = 51	*n* = 30
Mean ± SD, ARU	426 ± 45	427 ± 54	425 ± 30	637 ± 29
≥550 ARU *, n (%)	*4 (4%)*	*4 (7%)*	*0 (0%)*	*30 (100%)*
Multiplate^®^	*n* = 113	*n* = 61	*n* = 52	*n* = 30
ASPItest, Mean ± SD, AUC	97 ± 76	96 ± 82	98 ± 70	369 ± 153
ADPtest, Mean ± SD, AUC	337 ± 150	348 ± 164	325 ± 132	339 ± 128
COLtest, Mean ± SD, AUC	192 ± 105	215 ± 114	164 ± 87	455 ± 130

* Considered “aspirin resistant” by our laboratory definition.

**Table 3:** Platelet aggregation test results among post-stroke patients with clinical outcomes (death, recurrent stroke, heart attack, and/or amputation), *n* = 10.

Study ID No.	Outcome	Days from Index Stroke to Outcome	Platelet Aggregation Test			
			LTA AA (%)	LTA ADP (%)	LTA Collagen (%)	VerifyNow^®^ (ARU)
33	Death	260	15	50	21	427
44	Heart attack	1068	15	97 *	50	412
61	Recurrent stroke	837	9	74 *	26	422
99	Amputation	738	7	92 *	64 *	421
103	Recurrent stroke	44	12	75 *	22	405
111	Death	355	15	58	21	504
116	Recurrent stroke	611	14	71 *	45	440
129	Death	484	9	77 *	69 *	489
135	Heart attack	71	16	57	12	419
145	Recurrent stroke	155	11	62	31	414
	Mean ± SD	462 ± 347	12 ± 3	71 ± 15	36 ± 20	435 ± 34

* Considered “aspirin resistant” by definition: LTA AA ≥ 20%, LTA ADP ≥ 70%, LTA collagen ≥ 60%, or VerifyNow^TM^ ≥ 550 ARU.

## Data Availability

The data presented in this study are not publicly available and kept at the National Neuroscience Institute where the study was conducted.
